# A pharmacovigilance study of the association between linaclotide/plecanatide and muscle spasms based on food and drug administration adverse event reporting system

**DOI:** 10.3389/fphar.2025.1635792

**Published:** 2025-08-06

**Authors:** Xue Fan, Yuan Li, Xiangchun Lin

**Affiliations:** Department of Gastroenterology and Endoscopy Center, Peking University International Hospital, Beijing, China

**Keywords:** linaclotide, plecanatide, muscle spasms, pharmacovigilance, FAERS

## Abstract

**Background:**

Linaclotide and plecanatide, as guanylate cyclase-C agonists, are effective treatments for irritable bowel syndrome with constipation and chronic idiopathic constipation. While their therapeutic benefits are well-established, the potential association between these drugs and the adverse reaction of muscle spasms remains understudied and controversial.

**Aim:**

To evaluate the potential association between muscle spasms and linaclotide or plecanatide using real-world pharmacovigilance data.

**Method:**

Cases of muscle spasms linked to linaclotide or plecanatide as primary suspected drugs were extracted from the Food and Drug Administration Adverse Event Reporting System (FAERS). Disproportionality analyses, including reporting odds ratio and information component, were employed to detect safety signals. Temporal patterns were assessed using Weibull distribution analysis.

**Results:**

A total of 231 muscle spasms cases were identified (linaclotide: 182; plecanatide: 49). Females accounted for 72.3% of the cases (n = 167), indicating a higher susceptibility. Disproportionality analysis revealed significant safety signals for both drugs, with plecanatide showing a stronger association (ROR = 6.12, 95% CI: 4.61–8.11) compared to linaclotide (ROR = 1.88, 95% CI: 1.63–2.18). Weibull analysis demonstrated an early failure-type curve (β < 1), suggesting a higher incidence shortly after treatment initiation.

**Conclusion:**

This study identifies a significant association between linaclotide/plecanatide and muscle spasms. The findings highlight the need for vigilance in high-risk populations and call for further investigation into the underlying mechanisms.

## Introduction

Irritable bowel syndrome with constipation (IBS-C) and chronic idiopathic constipation (CIC) collectively affect approximately 20% of the general population, including a substantial proportion of elderly individuals, and significantly impair quality of life. As guanylate cyclase-C (GC-C) agonists, linaclotide and plecanatide are effective medications for various forms of chronic constipation, including IBS-C, idiopathic constipation, and functional constipation. Multiple clinical trials have confirmed their efficacy and safety in improving bowel habits and relieving abdominal symptoms in patients with CIC and IBS-C (Barish and Griffin, 2018; Brenner et al., 2024; Fukudo et al., 2018; Andresen et al., 2018; Pohl et al., 2019).

Drug-induced muscle spasms may lead to movement disorders, chronic pain, and metabolic complications such as rhabdomyolysis (Warren et al., 2002), requiring close monitoring of creatine kinase and electrolyte levels. Elderly patients face a significantly increased risk of falls, while those with neurological disorders are more susceptible to persistent spasticity (Tinetti et al., 1988; American Geriatrics Society, 2019, 2019). These spasms can severely limit mobility, worsen quality of life, and in severe cases potentially result in hospitalization or other serious complications.

The FDA Adverse Event Reporting System (FAERS) is a critical spontaneous reporting database that collects information on adverse event (AE), medication errors, and product quality complaints. It serves as a cornerstone of post-marketing pharmacovigilance. Disproportionality analysis, a common data mining approach applied to FAERS U.S. Food and Drug Administration (2025), enables the identification of potential drug-AE associations.

Given the widespread use and proven efficacy of linaclotide and plecanatide, understanding their adverse event profile is essential. To provide a comprehensive real-world safety assessment, a disproportionality analysis was performed using FAERS data to characterize potential associations between linaclotide/plecanatide and AEs, with a particular focus on the potentially underreported event of muscle spasms.

## Methods

### Data source

A retrospective pharmacovigilance study was conducted using data from the FAERS database dated from January 2004 to March 2025. Data mapping institutional review board approval for the present study was not required as the FAERS database is accessible to the public. The Medical Dictionary for Regulatory Activities were employed, the internationally recognized medical coding system developed by the International Council for Harmonisation of Technical Requirements for Pharmaceuticals for Human Use, for standardized terminology.

All of the adverse event terms and drug names were retrieved from the FAERS database. FAERS provides access to seven data categories, namely, demographic and administrative, drug information, adverse events, patient outcomes, report source, start and end dates of therapies, and therapeutic indications.

### Disproportionality analysis

As a validated approach in pharmacovigilance, disproportionality analysis served as a fundamental tool for detecting medication safety signals. The investigation implemented two established signal detection algorithms to evaluate linaclotide/plecanatide-related risks: the reporting odds ratio, and Bayesian confidence propagation neural network. Only adverse events that simultaneously exceeded all two methodological thresholds were classified as potential safety signals. All statistical computations were conducted with R programming language (v4.4.1).

In the 2 × 2 contingency table, cell “a” represented the number of reports associating the target drug with the target adverse drug reaction (ADR), “b” comprised reports of the target drug without the target ADR, “c” indicated reports of other drugs associated with the target ADR, while “d” represented reports involving neither the target drug nor the target ADR. Based on this framework, Table 1 presented the calculation formulas and decision criteria for both two signal detection algorithms (Hauben, 2003; Hauben et al., 2005).

**TABLE 1:** Formulae and criteria of two algorithms for signal detection.

Algorithm	Formula	Criteria
ROR	$\text{ROR}=\frac{\left(a/c\right)}{\left(b/d\right)}$ ; $95\%\text{CI}=e^{\ln\left(ROR\right)\pm 1.96\sqrt{\left(\frac{1}{a}+\frac{1}{b}+\frac{1}{c}+\frac{1}{d}\right)}}$	95% CI>1, a ≥2
BCPNN	$\text{IC}=\log_{2}\frac{a\left(a+b+c+d\right)}{\left(a+c\right)\left(a+b\right)}$ ; $\text{IC}025=e^{\ln\left(IC\right)-1.96\sqrt{\left(\frac{1}{a}+\frac{1}{b}+\frac{1}{c}+\frac{1}{d}\right)}}$	IC025 > 0

ROR, reporting odds ratio; BCPNN, Bayesian confidence propagation neural network; CI, confidence interval; IC, information component; IC025, the lower limit of the 95% two-sided CI of the IC.

### Weibull distribution analysis

The onset timing for linaclotide- and plecanatide-associated adverse reactions was calculated as the duration from treatment initiation to adverse event manifestation. Temporal patterns of adverse events were evaluated using Weibull distribution modeling.

The shape parameter (β) served as an indicator of hazard rate dynamics:

• β < 1 with 95% CI excluding 1: Early risk pattern (highest adverse event incidence shortly after treatment commencement, progressively declining)
• β ≈ 1 with 95% CI encompassing 1: Constant risk pattern (consistent adverse event probability during treatment)
• β > 1 with 95% CI excluding 1: Cumulative risk pattern (statistically significant increasing adverse event likelihood with prolonged therapy)

This analytical approach provides quantitative characterization of temporal risk profiles for these pharmacotherapies.

## Results

All FDA-approved indications of both drugs are summarized in Table 2. Linaclotide received initial FDA approval in 2012 (Kalola et al., 2024),followed by EMA in 2012 and Health Canada approval in 2013. Plecanatide was subsequently approved by the FDA in 2017 for the treatment of CIC.

**TABLE 2:** Summary of Marketing Start time of linaclotide and plecanatide.

Generic name	Brand name	Indication	Marketing start	Country
Linaclotide	Linzess Constella	chronic idiopathic constipation, functional constipation, irritable bowel syndrome with constipation	2012-08-30	the United States
			2012-11-26	EMA
			2013-12-03	Canada
Plecanatide	Trulance	chronic idiopathic constipation, irritable bowel syndrome with constipation	2017-01	the United States

**FIGURE 1:**
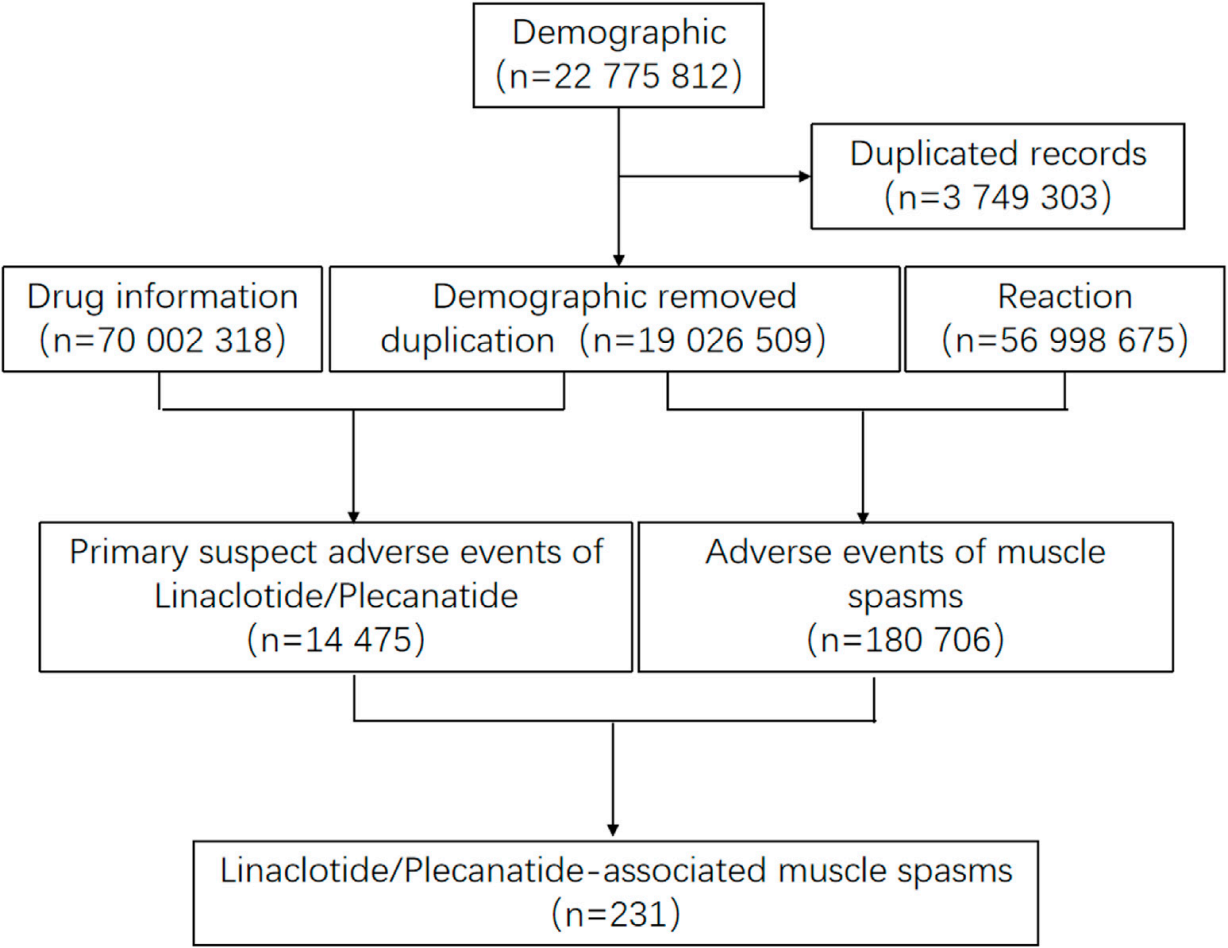
Process of the selection of cases of linaclotide/plecanatide associated muscle spasms from the FAERS database.

After screening 22,775,812 reports from FAERS, FDA guidelines were followed to remove duplicate entries by retaining only the most recent FDA_DT for records sharing the same CASEID. This process resulted in a final dataset of 19,026,509 reports eligible for further analysis (Figure 1).

### Descriptive characteristics

From 2013 to March 2025, 231 cases of muscle spasms were identified associated with linaclotide/plecanatide in the FAERS database, with clinical characteristics detailed in Table 3. These comprised 181 cases (78.4%) linked to linaclotide and 49 cases (21.2%) associated with plecanatide.

**TABLE 3:** Demographic and Clinical Characteristics of muscle spasms in patients receiving treatment with linaclotide/plecanatide.

Characteristics	Linaclotide (n = 182)	Plecanatide (n = 49)	Linaclotide/Plecanatide (n = 231)
Sex			
Female	141 (83.0%)	16 (32.7%)	167 (72.3%)
Male	27 (14.8%)	3 (6.1%)	30 (13.0%)
Missing	4 (2.2%)	30 (61.2%)	34 (14.7%)
Weight (kg)			
<50	7 (3.8%)	1 (2.0%)	8 (3.5%)
50∼100	31 (17.0%)	4 (8.2%)	35 (15.2%)
>100	0 (0%)	0 (0%)	0 (0%)
Missing	144 (79.1%)	44 (89.8%)	188 (81.4%)
Age (years)			
<18	0 (0%)	0 (0%)	0 (0%)
18∼64.9	38 (20.9%)	4 (8.2%)	42 (18.2%)
65∼85	45 (24.7%)	8 (16.3%)	53 (22.9%)
>85	7 (3.8%)	0 (0%)	7 (3.0%)
Missing	92 (50.5%)	37 (75.5%)	129 (55.8%)
Occupation			
Consumer	157 (86.3%)	27 (55.1%)	184 (79.7%)
Health Professional	1 (0.5%)	1 (2.0%)	2 (0.9%)
Physician	6 (3.3%)	18 (36.7%)	24 (10.4%)
Pharmacist	3 (1.6%)	2 (4.1%)	5 (2.2%)
Missing	15 (8.2%)	1 (2.0%)	16 (6.9%)
Reporter country			
Canada	1 (0.5%)	N/A	1 (0.4%)
Coutry not specified	2 (1.1%)	N/A	2 (0.9%)
Spain	2 (1.1%)	N/A	2 (0.9%)
United States	177 (97.3%)	49 (100%)	223 (97.8%)
Outcome			
Congenital Anomaly	0 (0%)	0 (0%)	0 (0%)
Death	0 (0%)	0 (0%)	0 (0%)
Disability	4 (2.2%)	0 (0%)	4 (1.7%)
Hospitalization	9 (4.9%)	0 (0%)	9 (3.9%)
Life-Threatening	0 (0%)	0 (0%)	0 (0%)
Other	169 (92.9%)	49 (100%)	218 (94.4%)

Of the cases, females and males accounted for 72.3% (n = 167) and 13.0% (n = 30), respectively. Most of the reports indicated that consumer, consisting of 79.7% (n = 184). The great majority of cases were from United States (n = 223) in terms of region, Canada (n = 1) and Spain (n = 2) in terms of country. A certain proportion of cases involved individuals aged 65-85 years, accounted for 22.9% (n = 53). There are 14.9% patients (n = 34) with records whose weights are distributed between 50 and 100 kg.

As shown in Table 4, based on the criteria for signal detection in each of the two algorithms, linaclotide/plecanatide were identified. Both signal detection algorithms identified a significant association between muscle spasms and linaclotide/plecanatide use. For linaclotide, Reporting Odds Ratio (ROR) was 1.88 (95% CI: 1.63-2.18), Information Component (IC) was 0.91 (IC025 0.70); for plecanatide, ROR was 6.12 (95% CI: 4.61-8.11), IC was 2.59 (IC025 2.18); combined ROR was 2.20 (95% CI: 1.94-2.51), and IC was 1.13 (IC025 0.94).

**TABLE 4:** Detected signals of muscle spasms of association between muscle spasms and linaclotide/plecanatide by two algorithms.

Regiman	a	ROR (95% two-sided CI)	IC(IC025)
Linaclotide	182	1.88 (1.63–2.18)	0.91 (0.70)
Plecanatide	49	6.12 (4.61–8.11)	2.59 (2.18)
Total	231	2.20 (1.94–2.51)	1.13 (0.94)

ROR, Reporting Odds Ratio; IC, Information Component.

**FIGURE 2:**
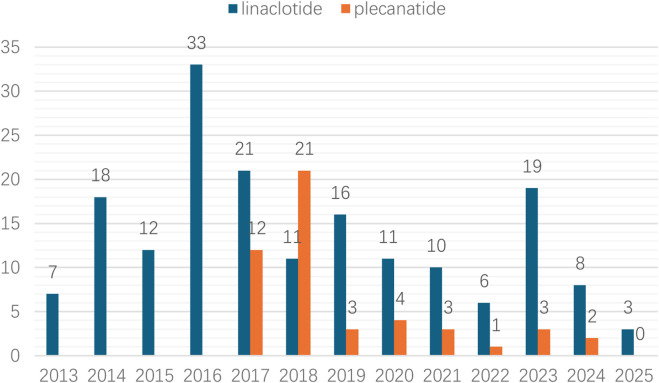
The annual reports on adverse reactions of the drugs linaclotide/plecanatide.

Linaclotide demonstrated a higher absolute number of adverse reaction cases compared to plecanatide. The annual case count for linaclotide-related muscle spasms gradually increased from 2013 to 2016, peaking at 33 cases (18.1%) in 2016. In contrast, plecanatide-associated cases peaked later in 2018 with 21 cases (42.9%), as illustrated in Figure 2.

**FIGURE 3:**
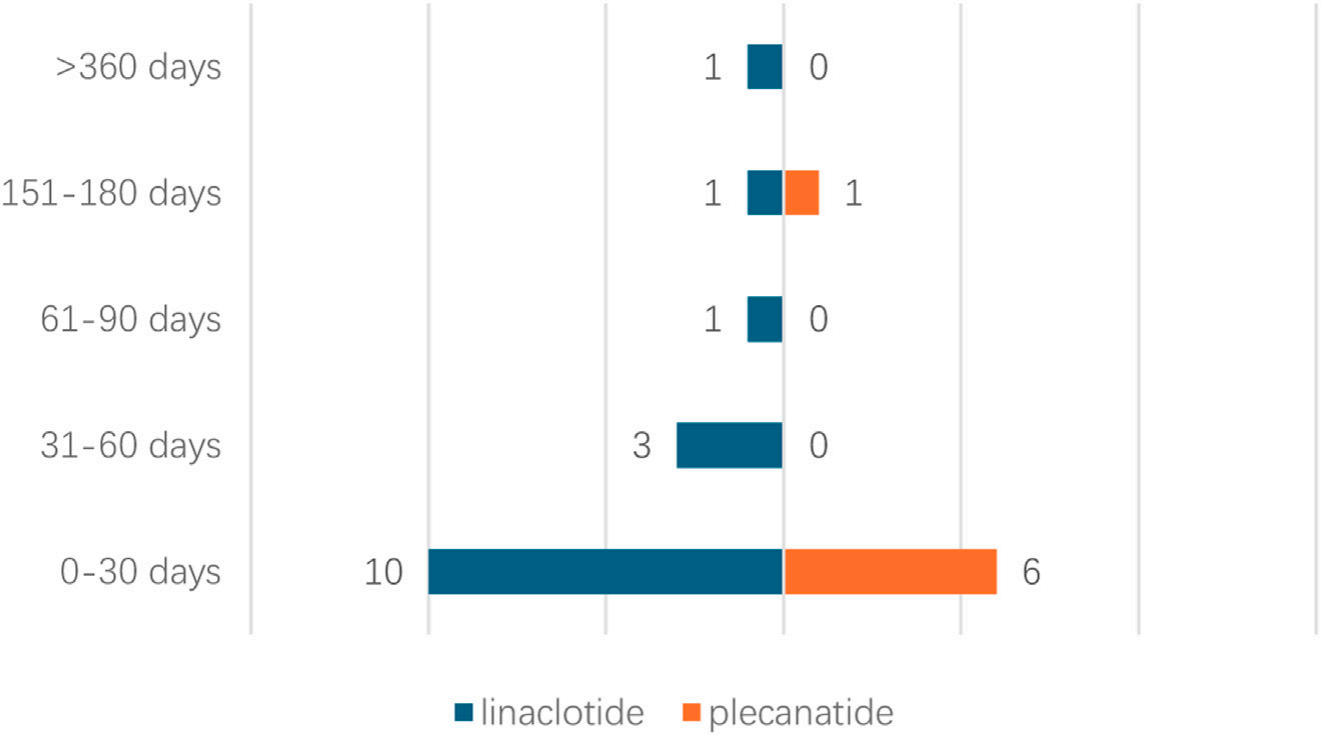
Time to onset analysis of adverse events associated with linaclotide/plecanatide.

Due to missing time information of onset time records of adverse events, the time to onset for 23 cases was analyzed. Time to onset analysis of adverse events associated with linaclotide/plecanatide is shown in Figure 3. 62.5% of linaclotide-associated events occurred within 30 days post-administration, 85.7% of plecanatide-associated events manifested within 30 days, One linaclotide-associated event was reported beyond 360 days.

A descriptive summary of the adverse reaction characteristics is shown in Table 5. The median time to adverse reaction onset differed between treatments, with linaclotide showing a longer duration (13.50 [IQR: 2.25, 46.25] hours/days) compared to plecanatide (2.00 [IQR: 1.00, 9.00]). The majority of participants were aged 18–64.9 years (39.1%), though a substantial proportion had missing age data (39.1%). Weight distribution showed 39.1% of patients in the 50–100 kg range, while sex was predominantly female (73.9%). Linaclotide was the more frequently administered drug (69.6% vs. plecanatide’s 30.4%). Serious adverse events (AEs) were reported in 26.1% of cases, with non-serious AEs comprising 73.9%. Missing data were notable for weight (56.6%) and sex (21.8%), suggesting potential limitations in data completeness.

**TABLE 5:** Descriptive statistics of adverse reaction onset time and related variables.

Characteristics	Category	Statistic/n (%)
Time	linaclotide	13.50 (2.25,46.25)
	plelcanatide	2.00 (1.00,9.00)
Age (years)	18-64.9	9 (39.1%)
	65-85	5 (21.8%)
	missing	9 (39.1%)
Weight (kg)	<50	1 (4.3%)
	50-100	9 (39.1%)
	missing	13 (56.6%)
Sex	female	17 (73.9%)
	male	1 (4.3%)
	missing	5 (21.8%)
drugs	linaclotide	16 (69.6%)
	plelcanatide	7 (30.4%)
Serious AE	yes	6 (26.1%)
	no	17 (73.9%)

In the Weibull distribution analysis of FAERS data, a shape parameter 0.453,0.563 and 0.459, suggests that ADRs are more likely to occur early in treatment (e.g., allergic reactions). Adverse drug reactions (ADRs) predominantly occur early in treatment. This pattern resembles the “early failure-type” curve seen in allergic reactions. The decreasing incidence rate over time indicates a susceptible population experiencing events during initial treatment phases,as detailed in Table 6.

**TABLE 6:** Weibull shape parameter test for muscle spasms associated with linaclotide/plecanatide.

Database	Scale parameter:α (95% CI)	Shape parameter:β (95% CI)	Type
Linaclotide	20.22 (16.63–23.80)	0.453 (0.430–0.477)	Early failure
Plecanatide	7.773 (4.700–10.85)	0.563 (0.482–0.644)	Early failure
Linaclotide/plecanatide	18.33 (15.29–21.36)	0.459 (0.434–0.478)	Early failure

## Discussion

Linaclotide and plecanatide, as guanylate cyclase-C (GC-C) agonists, represent important therapeutic options for chronic constipation disorders. Their mechanism of action involves binding to transmembrane GC-C receptors to promote chloride/bicarbonate secretion while inhibiting sodium absorption, thereby enhancing intestinal fluid content and accelerating gastrointestinal transit (Layer and Stanghellini, 2014). The most commonly reported adverse event associated with both drugs is diarrhea, occurring at an incidence rate of approximately 4%∼15.4% (Barish and Griffin, 2018; Fukudo et al., 2018; Andresen et al., 2018; Pohl et al., 2019). This study provides the first comprehensive evaluation of muscle spasm events associated with these medications in real-world clinical practice.

There are two synonyms for “muscle spasms”, which are “muscle cramp” and “muscle spasms”, yet their distinguishing boundaries are unclear. “Muscle cramp” usually refers to a sudden and painful contraction of the muscle, which is generally caused by muscle fatigue, electrolyte imbalance (such as potassium or calcium deficiency), overuse of the muscle and so on. The meaning of “muscle spasms” is broader. Besides including the similar meaning of sudden contraction as “muscle cramp”, it can also be used to describe involuntary and repeated contractions of the muscle due to nervous system problems, diseases and other factors. They may be conflated and represent partially overlapping phenomena (Therkildsen et al., 2024). Skeletal muscle cramps often take place when it comes to pregnancy, advanced age, exercise or motor neuron disorders. It has been reported in the papers that some drugs may cause muscle cramps. Sonic Hedgehog Inhibitors, in Basal Cell Carcinomas (Nguyen et al., 2023),the most common adverse effects for vismodegib and sonidegib were muscle spasms. The author took into account the association with serotonin toxicity. Furthermore, anesthetic drugs, including thiopentone and suxamethonium as early as the 1980s, there were clear report on medical cases of severe muscle spasms (Mostafa and Virdee, 1985). There were also reports in addition, such as propofol, etomidate (Hao et al., 2020). Other medications, such as Paroxetine (Mostel et al., 2022), quetiapine (Mostel et al., 2022), can also cause muscle cramps.

The study specifically examined reports coded as “muscle spasms” in the FAERS database, which does not differentiate between skeletal muscle spasms and smooth muscle spasms. This limitation should be considered when interpreting the findings. Three potential mechanisms are reported through which potential mechanisms underlying the association between linaclotide/plecanatide and muscle spasms:

Firstly, direct drug effects may be a proposed mechanism. Linaclotide/plecanatide themselves can activate the cystic fibrosis transmembrane conductance regulator (CFTR), resulting in increased intestinal fluid and accelerated gastrointestinal transit to speed up bowel movement, while at the same time, they can increase levels of extracellular cGMP in the submucosa to inhibit colonic nociceptors, thereby relieving intestinal pain (Castro et al., 2013). Based on the above mechanistic analysis, linaclotide may be associated with smooth muscle spasms. Unlike linaclotide, the mechanism mentioned in plecanatide’s instructions indicates that it reduced abdominal muscle contractions, a measure of intestinal pain. The mechanism has not been studied. This may be associated with differential intestinal responses elicited by the two drugs.

Subsequently, the dehydration and electrolyte imbalance theory is the oldest theory. Exercise-associated muscle cramps patients may have a higher sweating rate and sodium loss (Miller et al., 2022). Conversely, there are also some studies that do not support the above-mentioned old theories (Schwellnus, 2009; Braulick et al., 2013; Casa et al., 2015; Hoffman et al., 2015; Szymanski et al., 2022; Garrison et al., 2020). Patients undergoing hemodialysis who experience muscle cramps also have electrolyte imbalance mechanisms mentioned. Hypomagnesemia can lead to muscle spasms through mechanisms such as affecting the concentrations of sodium, potassium and calcium ions in cell membranes and regulating the renal outer medullary potassium channels located in the distal nephron (Varghese et al., 2020). Moreover, some scholars have reported incidents of muscle spasms in hemodialysis patients, they inferred that activation of the sympathetic nervous system during hemodialysis was responsible for reducing intracapillary pressure to the extent that capillary derecruitment led to skeletal muscle ischemia and cramping (Atkinson, 2019). Linaclotide and plecanatide’s mechanisms of action include: CFTR activation promotes the secretion of chloride and bicarbonate ions and inhibits sodium absorption (Kalola et al., 2024; Layer and Stanghellini, 2014). Then the two drugs may increase sodium/chloride ion secretion, potentially causing secondary hyponatremia or hypomagnesemia, elevated muscle excitability or spasms. Therefore, the theory of sodium loss may support the mechanism by which the aforementioned drugs.

The third mechanism is the neuro-reflex hypothesis. Current research suggests that intestinal stimulation may induce muscle spasms through the viscero-somatic neuro-reflex pathway. Grundy and Schemann (Spencer and Hu, 2020)demonstrated that chemical or mechanical stimulation of the gut can activate the central nervous system via vagal/spinal afferent pathways, leading to hyperexcitability of motor neurons. Mayer et al. (Mayer et al., 2015) further elucidated the role of the gut-brain axis, proposing that gut-derived signals transmitted via the vagus nerve to the nucleus tractus solitarius may subsequently influence motor neuron pool activity in the spinal cord. Clinical studies by Farmer and Aziz (Farmer and Aziz, 2013) revealed that intestinal inflammation or chemical stimulation in animal models can trigger somatic muscle contractions through the spinothalamic pathway. Collectively, these studies indicate that GC-C agonists may alter the local intestinal environment, activate neuro-reflex pathways, and consequently lead to adverse effects such as muscle spasms.

Although linaclotide and plecanatide are both GC-C agonists, there are subtle differences between them. Plecanatide structurally mimics the endogenous hormone uroguanylin, with its activity regulated by intestinal pH levels. Its effects are concentrated in the proximal small intestine, resulting in less stimulation of the distal colon, theoretically leading to a lower risk of diarrhea. From the electrolyte imbalance hypothesis perspective, plecanatide’s high activity in the proximal small intestine may increase sodium/chloride ion secretion, potentially causing secondary hyponatremia or hypomagnesemia, elevated muscle excitability spasms. From the neuro-reflex hypothesis perspective, excessive peristalsis in the proximal small intestine may trigger muscle spasms through viscero-somatic neural reflexes. The study results indeed demonstrate that plecanatide’s ROR = 6.12 (vs. linaclotide’s 1.88) suggests a significantly higher risk of muscle spasms, which may be associated with its specific distribution pattern as shown in Table 4. Consequently, plecanatide needs a closer clinical monitoring. While linaclotide carries lower risk, vigilance for spasm symptoms remains essential during long-term therapy. Clinical decisions should incorporate individual risk stratification, including electrolyte imbalance history and pre-existing neurological conditions. Additionally, the later market introduction of plecanatide (2017) compared to linaclotide (2012) may contribute to its relatively lower number of reported adverse reactions, as post-marketing surveillance data accumulate more slowly for newer medications.

Analysis revealed notable gender differences in reported muscle spasm events. Female patients accounted for 72.3% of cases during treatment with linaclotide/plecanatide, suggesting a higher reporting frequency of this association in females. Several factors may explain this observation. First, electrolyte metabolism differences. Estrogen may increase potassium excretion by suppressing the renin-angiotensin system (Beland et al., 1994), while postmenopausal women show an average reduction in serum calcium levels. Second, hormonal fluctuations. Elevated progesterone during the luteal phase may promote extracellular potassium shifts. Menstrual cycle-associated magnesium loss may lower the muscle excitation threshold (Li et al., 2001). Third, reporting bias. Females tend to report nonspecific symptoms (e.g., muscle spasms) more frequently. The prevalence of chronic constipation and IBS-C is significantly higher in female patients compared to males. Expert consensus documents (Andresen et al., 2007; Suares and Ford, 2011) ^i^dentify women as the primary population with unmet therapeutic needs in IBS management. This gender disparity in baseline disease prevalence aligns with findings from multiple multicenter prospective studies (Brenner et al., 2024; Zhou et al., 2024; Quigley et al., 2013) that consistently show female predominance in constipation-predominant functional bowel disorders. Nevertheless, gender-matched controlled trials failed to yield statistically significant findings on potential sex disparities. Therefore, these findings may primarily reflect underlying epidemiological characteristics rather than true pharmacological differences. As documented in the clinical research literature (Lembo et al., 2010), women generally demonstrate greater willingness and tendency to participate in clinical research than men and exhibit better compliance with physicians’ requirements to complete trials and generate valid results. Previous phase I safety and pharmacodynamic studies of linaclotide were conducted predominantly in male populations, demonstrating that linaclotide is safe and well-tolerated. Previous studies (Rao et al., 2023) have specifically investigated the demographic characteristics of plecanatide-treated patients. The findings indicated that plecanatide exhibited comparable safety profiles to placebo across all age and BMI subgroups. However, like most clinical trials in this therapeutic area, the reported adverse events focused on gastrointestinal symptoms (diarrhea, abdominal pain) and general complaints (headache), with no specific mention of muscle spasms - highlighting the value of post-marketing surveillance in detecting less common adverse effects.

Comparative analysis of adverse reaction timelines revealed distinct patterns between the two drugs. Linaclotide was associated with higher initial reporting rates of adverse events with subsequent fluctuations, suggesting early intolerance followed by variable adaptation. Whereas plecanatide exhibited the classic declining-risk profile typically seen with novel therapeutics, indicating progressive development of tolerability over time. These divergent patterns likely stem from multiple factors including differential medication histories, distinct target populations, and mechanistic variations in receptor binding kinetics. Both agents shared favorable long-term safety profiles (Table 6) while displaying characteristic early-failure patterns (β < 1), with linaclotide’s significantly higher α value (20.22 vs. 7.773) indicating delayed failure onset compared to plecanatide. These temporal characteristics have important clinical implications: (1) heightened vigilance during initial treatment cycles is essential for both drugs, particularly the first 4 weeks; (2) therapeutic selection should consider linaclotide for sustained long-term regimens due to its prolonged effectiveness, while reserving plecanatide for short-term interventions or as an alternative option given its quicker tolerance establishment. Prospective comparative studies with incidence-based endpoints are warranted to validate these temporal patterns and elucidate their underlying mechanisms, particularly through PK-PD modeling approaches that could inform risk-stratified treatment protocols. Therefore, close monitoring is advised during the first 4 weeks of treatment with either medication.

When interpreting findings from studies utilizing spontaneous reporting databases, several potential limitations should be considered. First, the FAERS does not contain data on all linaclotide/plecanatide treatment and events in the world, nor does it document all adverse events that occur in clinical practice. Underreporting of adverse events may lead to underestimation of the true association. Second, the database contains incomplete data entries, with missing or inconsistent information across reports. Missing clinical details (e.g., comorbidities, concomitant medications) hinder confounder adjustment. The absence of denominator data precludes calculation of incidence rates. Third, the proposed mechanisms remain speculative and require experimental validation. These limitations necessitate cautious interpretation of the signals as hypothesis-generating rather than definitive evidence. These limitations highlight the need for additional research to explore the specific mechanisms by which linaclotide/plecanatide might associated withmuscle spasms, including controlled clinical studies, basic science investigations of drug effects on neuromuscular function, and more detailed analyses of risk factors predisposing certain patients to these adverse effects. Such research would help clinicians better predict, prevent, and manage these potentially debilitating medication side effects.

## Conclusion

This study identifies a significant association between linaclotide/plecanatide use and the occurrence of muscle spasms, with plecanatide showing a stronger signal of association based on FAERS data. The literature reports indicate that potential mechanisms that may explain the association include direct activation of intestinal receptors, electrolyte imbalances and neuro-reflex activation. Notably, the adverse events predominantly occurred during the early treatment phase and were more frequently reported in female patients. These results highlight the need for vigilance in high-risk populations. Further mechanistic researches and prospective pharmacovigilance efforts are warranted to substantiate these associations and guide risk-mitigation strategies.

## Data Availability

The original contributions presented in the study are included in the article/supplementary material, further inquiries can be directed to the corresponding author.
